# Intrinsic Properties of Multi-Layer TiO_2_/V_2_O_5_/TiO_2_ Coatings Prepared via E-Beam Evaporation

**DOI:** 10.3390/ma15113933

**Published:** 2022-05-31

**Authors:** Irfa Rehman, Muhammad Bilal Hanif, Abdulaziz Salem Alghamdi, Abdul Khaliq, K. S. Abdel Halim, Tayyab Subhani, Martin Motola, Abdul Faheem Khan

**Affiliations:** 1 Department of Materials Science and Engineering, Institute of Space Technology, 1-National Highway, Islamabad 44000, Pakistan; effiaffi92@gmail.com; 2 Department of Inorganic Chemistry, Faculty of Natural Sciences, Comenius University in Bratislava, Ilkovicova 6, Mlynska Dolina, 842 15 Bratislava, Slovakia; hanif1@uniba.sk; 3 College of Engineering, University of Ha’il, Ha’il P.O. Box 2440, Saudi Arabia; a.alghamdi@uoh.edu.sa (A.S.A.); ab.ismail@uoh.edu.sa (A.K.); k.abdulhalem@uoh.edu.sa (K.S.A.H.); 4 Central Metallurgical Research and Development Institute (CMRDI), P.O. Box 87, Helwan 11421, Egypt

**Keywords:** TiO_2_, V_2_O_5_, multi-layer, thin films, Rutherford backscattering (RBS), solar water heater

## Abstract

Nanocomposite multi-layer TiO_2_/V_2_O_5_/TiO_2_ thin films were prepared via electron-beam evaporation using high-purity targets (TiO_2_ and V_2_O_5_ purity > 99.9%) at substrate temperatures of 270 °C (TiO_2_) and 25 °C (V_2_O_5_) under a partial pressure of oxygen of 2 × 10^−4^ mbar to maintain the stoichiometry. Rutherford backscattering spectrometry was used to confirm the layer structure and the optimal stoichiometry of the thin films, with a particle size of 20 to 40 nm. The thin films showed an optical transmittance of ~78% in the visible region and a reflectance of ~90% in the infrared. A decrease in transmittance was observed due to the greater cumulative thickness of the three layers and multiple reflections at the interface of the layers. The optical bandgap of the TiO_2_ mono-layer was ~3.49 eV, whereas that of the multi-layer TiO_2_/V_2_O_5_/TiO_2_ reached ~3.51 eV. The increase in the optical bandgap was due to the inter-diffusion of the layers at an elevated substrate temperature during the deposition. The intrinsic, structural, and morphological features of the TiO_2_/V_2_O_5_/TiO_2_ thin films suggest their efficient use as a solar water heater system.

## 1. Introduction

Economic growth and the everyday activities of human life are dependent on energy. However, the depletion of fossil fuels and environmental factors such as global warming have made the energy crisis of paramount importance, necessitating a search for more dependable means to fulfill energy requirements. The technology for harvesting renewable energy is still being developed, as it has not yet reached the required standard. As solar energy is a prominent source of renewable energy, the aim of this paper is to improve the efficiency of energy-harvesting devices. Solar energy can be converted into electrical and thermal energy via the photovoltaic effect, and materials based on this phenomenon are now used in solar cells and solar heaters [1]. The efficient utilization of incident light is crucial for a photovoltaic cell’s performance, i.e., the generation of the charge carriers (e^−^/h^+^). The photogenerated charge carriers are subsequently transported to the electrodes that facilitate the flow of e^−^/h^+^, converting them to current. Such energy is currently used in industrial and domestic facilities (the operation of machinery, air conditioning, and lighting) [2]. In the case of solar heaters, the incident photons are converted to useable heat via the so-called solar–thermal energy conversion. In solar–thermal devices, the photonic radiation is converted to phononic vibration in the absorber, which is then transported to the desired location using a carrier material (e.g., water). The applications of this process include space heating, industrial processes (generation of heat), air conditioning, water heating, drying, desalination, distillation, and electrical energy generation [3,4].

Three crucial intrinsic properties of a solar water heater device (SWHD) are required to maximize its efficiency: (1) good thermal conduction, (2) incident light utilization, and (3) high transmittance [5,6]. In addition, low-reflective materials are important for efficient SWHDs, as reflected incident light is considered a loss in terms of energy, thus decreasing the performance of SWHDs [7]. Moreover, SWHDs generate temperatures of up to 1000 °C; thus, high thermal stability is essential [4].

Vanadium oxide (V_2_O_5_) is a chromogenic material that has been studied extensively due to its unique photoelectrochemical properties [8,9,10]; nowadays, it is used in energy-efficient smart windows [11]. Bulk V_2_O_5_ undergoes a semiconductor-to-metallic phase transition at 257 °C, possessing an orthorhombic structure with an indirect bandgap of 2.6 eV [12,13]. At high temperatures (>Tc), the metal-like V_2_O_5_ possesses a different crystal structure compared to that at low temperatures. At room temperature, it possesses an orthorhombic structure which transforms to a metastable monoclinic structure at around 450 °C [14]. Nevertheless, Kang M. et al. [15] reported V_2_O_5_ thin films without any crystal structure or phase transition changes during the insulator-to-metal transition of V_2_O_5_. Indeed, the phase transition corresponds to a change in the electrical conductivity (with changes of up to 10 orders of magnitude), whereas discontinuity can be seen for the optical and magnetic properties of V_2_O_5_ [16].

The solar water heater system proposed in this study constitutes multi-layer TiO_2_ and V_2_O_5_ thin films. V_2_O_5_ has variable optical properties and thus has been used in diverse technological applications such as infrared (IR) detectors, memory devices, smart windows, artificial muscles, electronic information displays, lithium batteries, and optical and electrical switches [17,18,19,20,21,22,23]. There are several oxides of vanadium, each with stability over a particular composition range. The thin films of vanadium pentoxide show a refractive index of 1.9–2.09 [24], with a bandgap value range of 2.04–3.25 eV [24,25,26,27]. This high refractive index is associated with good transparency in the visible region.

The bandgap of TiO_2_ is wide, and thus it finds its way into various applications, including photocatalysis, optical fibers, photo electrolysis, biomedical processes, and photovoltaics [15,28,29,30]. TiO_2_ has been diversely employed in solar devices in the form of cathodes, light scatterers, electron collectors, high wavelength transmitters, etc. These applications are possible due to its tunable conductivity, resistance to weathering, self-cleaning ability, excellent transmittance of solar radiation, and ease of fabrication [31,32,33,34,35,36,37,38,39,40,41,42,43,44,45,46,47]. TiO_2_ has been utilized in the form of a mesoporous nanostructure to increase scattering, which increases the interaction of light with absorbing materials and eventually improves the absorption efficiency [31]. The three crystalline forms of TiO_2_ have different crystal structures, and thus their optical properties also differ [32]. The anatase phase of TiO_2_ thin films is promising for applications in optics because of its high transparency in the visible region and its high reflective index [33]. Furthermore, V_2_O_5_ films along with TiO_2_ have been prepared using the electron beam evaporation technique due to its cost-effectiveness, easy control of parameters, uniformity, and stoichiometry, which is still a challenge in other PVD fabricated films. However, the stoichiometry of the system can be controlled easily by the annealing process. Comparatively, sputtering, atomic layer deposition, and molecular beam epitaxy produce more stoichiometric films than the e-beam technique; however, these are expensive techniques and ultimately increase the cost of the whole system. An additional advantage of the e-beam technique is that we can achieve a higher deposition rate without affecting the uniformity and stoichiometry of the films.

In the present work, we prepared SWHDs based on a nanocomposite TiO_2_/V_2_O_5_/TiO_2_ multi-layer thin film via e-beam evaporation [48,49], consisting of a ~50 nm-thick V_2_O_5_ layer sandwiched between two TiO_2_ layers (a ~100 nm-thick bottom layer and a ~360 nm-thick upper layer) on a transparent 1 mm-thick soda–lime glass (as depicted in the graphical abstract). A comprehensive characterization of the intrinsic properties of the TiO_2_/V_2_O_5_/TiO_2_ SWHD was conducted using X-ray diffractometry (XRD), scanning electron microscopy (SEM), energy-dispersive X-ray spectroscopy (EDS), Rutherford backscattering spectrometry (RBS), atomic force microscopy, and transmittance spectra (TS). The prepared TiO_2_/V_2_O_5_/TiO_2_ thin film possessed suitable properties for potential application in solar-to-heat conversion, i.e., SWHDs.

## 2. Experimentation

TiO_2_ (purity > 99.99%) and V_2_O_5_ (purity > 99.9%) powders were used as starting materials, which were converted to pellets using polyvinyl gel (PVA) as a binder. Briefly, 2.5 g of PVA gel was dissolved in 100 mL of DI H_2_O. Afterward, the solution (binder) was heated to 200 °C and stirred for 3 h. The binder solution was subsequently used for the preparation of 10 mm-thick pellets by applying a 799.934 mbar hydraulic press.

A nanocomposite TiO_2_/V_2_O_5_/TiO_2_ multi-layer thin film was fabricated using the electron beam (e-beam) evaporation technique. The deposition was performed under a vacuum of 1 × 10^−5^ mbar and the partial pressure of oxygen was kept to 2 × 10^−4^ mbar during the deposition of TiO_2_ (deposition rate ~0.45 nm/s) and V_2_O_5_ (deposition rate ~0.15 nm/s) to maintain the stoichiometry of the layers. The first layer of TiO_2_ (~100 nm thickness) was deposited on a soda–lime glass substrate at a substrate temperature of 270 °C. Subsequently, the second layer of V_2_O_5_ (~50 nm thickness) was deposited at a substrate temperature of 25 °C. Lastly, the third layer of TiO_2_ (~360 nm thickness) was deposited at a substrate temperature of 270 °C (the device is visualized in the graphical abstract). To distinguish the different layered structures, we refer to them as follows: (i) mono-layer, i.e., 100 nm-thick TiO_2_; (ii) bi-layer, i.e., 50 nm-thick V_2_O_5_ on 100 nm-thick TiO_2_; and (iii) tri-layer, i.e., 360 nm-thick TiO_2_ on 50 nm-thick V_2_O_5_ on 100 nm-thick TiO_2_.

The stoichiometric analysis and the thickness of each layer was measured with Rutherford backscattering spectroscopy (RBS) using a 2MeV Pelletron Tandem Accelerator (5UDH-2 Pelletron). The mean energy used for the RBS analysis was 2 MeV (He^2+^ beam). The incident and scattering angles were 70° and 170°, respectively. Cornell geometry was used for the measurements, with a constant angle of 170° and 13 cm of distance between the sample and the detector. The simulation software SIMNRA was used for the data analysis.

Field-emission scanning electron microscopy (FESEM, MIRA3 TESCAN) and energy-dispersive X-ray spectroscopy (EDS) were used to determine the surface morphology, topography, and elemental properties of the films.

An X-ray diffractometer (XRD, PANalytical, Cu Ka radiation, λ = 1.5418 Å) was used to study the crystal structure of the nanocomposite at room temperature.

To obtain deeper insight into the influences of the surface roughness and skewness on the different TiO_2_ nanostructures, atomic force microscopy (AFM, Quesant Universal SPM, Ambios Technology) was conducted in contact mode using standard silicon AFM probes.

The electrical resistivity of the film was analyzed using a DC-4-point probe method at 25 °C.

Optical transmittance was measured at 25 °C by a Perkin Elmer UV/VIS/NIR Lambda 19 spectrophotometer in the wavelength range of 250–2500 nm.

## 3. Results and Discussion

**Figure 1:**
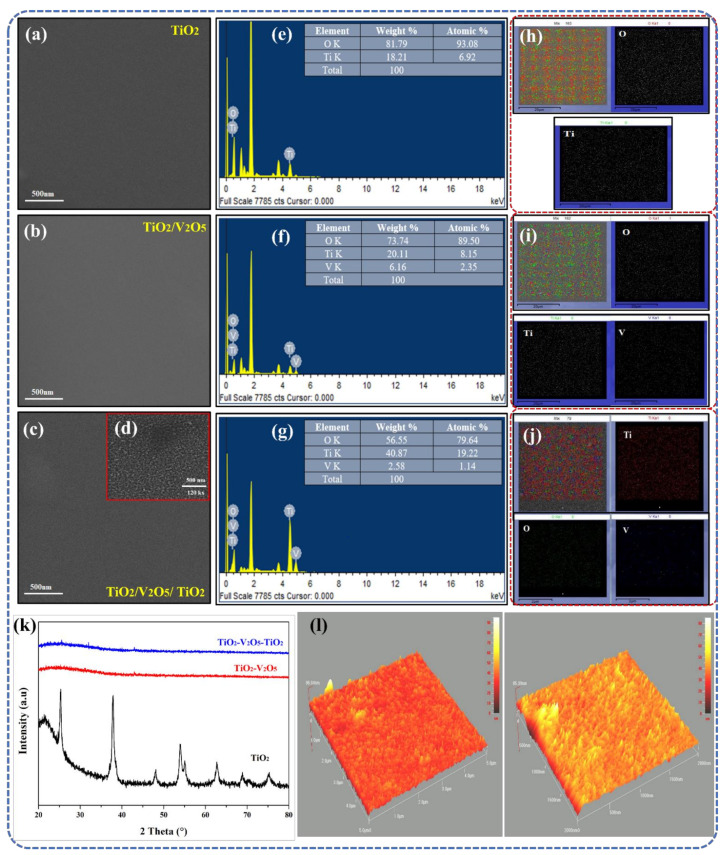
Surface morphology of (**a**) TiO_2_, (**b**) TiO_2_/V_2_O_5_, and (**c**) TiO_2_/V_2_O_5_/TiO_2_ at 80 kx magnification and (**d**) TiO_2_/V_2_O_5_/TiO_2_ at 120 kx magnification. EDS analysis and elemental area mapping of (**e**,**h**) single-layer TiO_2_, (**f**,**i**) bi-layer TiO_2_/V_2_O_5_, and (**g**,**j**) multi-layer TiO_2_/V_2_O_5_/TiO_2_ thin film. (**k**) XRD analysis of single-, bi-, and tri-layer thin films. (**l**) AFM topography of a multi-layer TiO_2_/V_2_O_5_/TiO_2_ thin film on 2 × 2 μm^2^ and 5 × 5 μm^2^, respectively.

First, a step-by-step FESEM analysis (layer-by-layer) was conducted to determine the surface morphology of our SWHD. The top views of the mono-, bi-, and tri-layers are depicted in Figure 1a–c. In general, the surfaces of all the layers showed dense, smooth structures with good uniformity, i.e., no cracks or peel-offs were present. At higher magnifications of the tri-layer (Figure 1d), a nanocomposite surface was observed with a clear nanoparticle-like structure and a particle size in the range of 20–40 nm. The quality of the film improved with an increase in the substrate temperature during deposition, as there was an adequate amount of energy for atoms to be arranged uniformly.

EDS mapping was conducted to determine the presence and distribution of elements across the whole surface. The results are summarized in Table 1. The energy dispersive X-ray spectroscopic (EDS) analysis of the single-layer TiO_2−_ film is shown in Figure 1e. EDS revealed the presence of Ti atoms in the film. The glass substrate contributed to the excessive oxygen in the films. Figure 1f shows the EDS analysis of the TiO_2_/V_2_O_5_ bi-layer film, which revealed the presence of vanadium atoms alongside the Ti atoms, indicating the development of the TiO_2_/V_2_O_5_ bi-layer film. The EDS analysis of the TiO_2_/V_2_O_5_/TiO_2_ tri-layer film is shown in Figure 1g. The Ti atoms are present in greater amounts than in the EDS results in Figure 1e,f, indicating the formation of the TiO_2_/V_2_O_5_/TiO_2_ tri-layer film.

Elemental area mapping was also performed to confirm the results of EDS. These results are shown in Figure 1h–j, and the relative decrease in oxygen atoms due to the addition of V and Ti atoms is depicted in Figure 1i,j. The deposition and dispersion of single-layer, bi-layer, and multi-layer thin films were uniform throughout the surface, as characterized by the results of the elemental area mapping. Overall, the EDS results corresponded well with those of the elemental area mapping, indicating that the TiO_2_/V_2_O_5_/TiO_2_ tri-layer film was effectively fabricated.

As reported [4], a high thermal stability (up to 1000 °C) is necessary for a material to be potentially used as a SWHD. Thus, a simple test was conducted in order to evaluate the thermal stability of our TiO_2_/V_2_O_5_/TiO_2_ tri-layer film. The sample was put in a muffle oven and heated up to 1000 °C for 3 h. No cracks were visible on the surface of the TiO_2_/V_2_O_5_/TiO_2_ tri-layer film, indicating that a good thermal stability was achieved.

Figure 1k shows the XRD patterns for the mono-, bi-, and tri-layers. In the case of the TiO_2_ mono-layer, only one crystal structure was identified: tetragonal anatase TiO_2_ (space group P4_2_/mnm, ICCD 01-086-1157) [50]. After depositing an additional layer (V_2_O_5_ in the bi-layer and V_2_O_5_/TiO_2_ in the tri-layer), the amorphous nature of the thin film was identified. Using the Scherer formula, the crystallite size was determined to be 23.5 nm for the single-layer TiO_2_ film. As previously reported, this is a well-known behavior of such thin films [51,52]. All in all, the proposed tri-layer thin film is suitable for use as a SWHD due to its promising surface morphology and crystal structure.

Additional information about the surface morphology alterations of the TiO_2_/V_2_O_5_/TiO_2_ tri-layer film was acquired using AFM (Figure 1l) The relevant nanoscale roughness was determined on areas of 2 × 2 μm^2^ and 5 × 5 μm^2^, respectively. Such AFM scans are considered representative and are beneficial for studying the surfaces of thin films [51]. Furthermore, surface roughness also describes the light scattering along with the quality of the surface under investigation. The surface of the TiO_2_/V_2_O_5_/TiO_2_ tri-layer film was observed to have no cracks and to be rather smooth (with low roughness values), which is necessary for the thin film to be considered for application as a SWHD.

**Figure 2:**
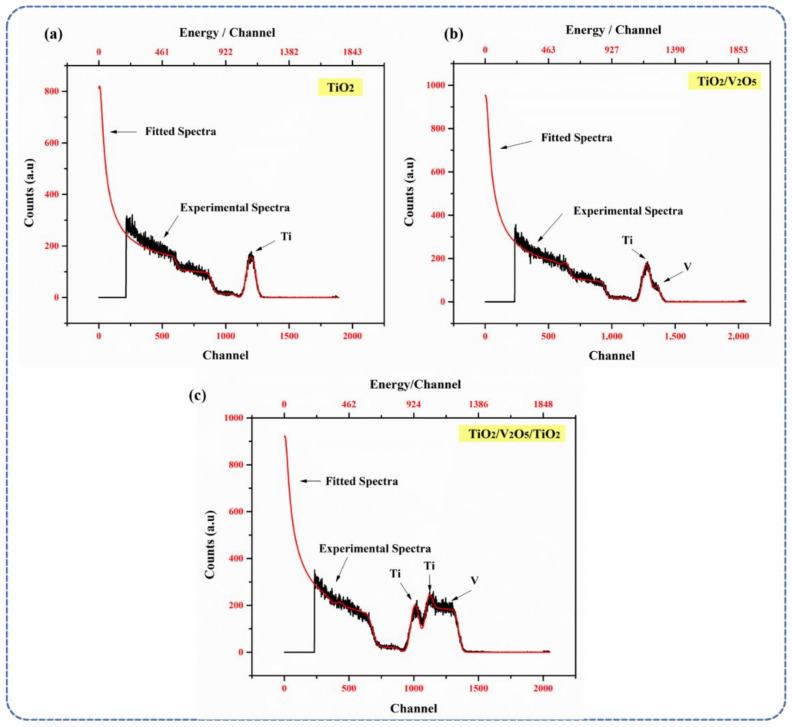
RBS spectra of (**a**) mono-layer TiO_2,_ (**b**) bi-layer TiO_2_/V_2_O_5_, and (**c**) multi-layer TiO_2_/V_2_O_5_ thin films, respectively.

The RBS spectra of the mono-, bi-, and tri-layers are shown in Figure 2a–c. Only one peak was observed in the mono-layer (Figure 2a), which confirms the presence of TiO_2_ and the thickness (150 nm) of the layer, with a Ti:O atomic ratio of 30%:70%. The RBS spectra of the bi-layer (Figure 2b) showed two peaks representing Ti (Ti 30%: O_2_ 70%) and V (V 12%: O_2_ 88%), respectively. The kinematic factors for Ti and V are 0.7166 and 0.7313, respectively; thus, the diffusion of the peaks was observed [34,35]. The RBS spectra of the tri-layer (the final concept of our SWHD) showed one peak for V and two peaks for Ti. The 100 nm-thick bottom TiO_2_ layer was observed at a higher channel (I_max_ ~ 1100 channel) compared to that of the 360 nm-thick top TiO_2_ layer (I_max_ ~ 1000 channel). The position of the I_max_ was kinematic factor-dependent, i.e., the lower TiO_2_ layer appeared at a higher channel. The 50 nm-thick V_2_O_5_ layer (I_max_ ~ 1250 channel) was sandwiched between the two TiO_2_ layers. The RBS data confirmed the formation of a multi-layered structure within the tri-layer, with optimal stoichiometry and film thickness (Table 1). The calculated thicknesses of the layers differ from the intended (theoretical) ones as follows: (i) 150 nm-thick bottom TiO_2_ layer (theoretical: 100 nm); (ii) 54 nm-thick V_2_O_5_ layer (theoretical: 50 nm); and (iii) 400 nm-thick top TiO_2_ layer (theoretical: 360 nm). The following equation was used to determine the thicknesses of the layers via RBS, where the total energy loss (Δ*E*) is proportional to the depth (*t*). With this approximation, the relationship between the energy width Δ*E* of the signal from the thickness of the film (Δ*t*) can be derived:

$$\Delta E=\Delta t\left(k\frac{dE}{dx_{in}}+\frac{1}{cos\theta }\frac{dE}{dx_{out}}\right)$$

where *θ* is the lab scattering angle, *k* is the kinematic factor, and *x_in_* and *x_out_* indicate the energies at which the rate of energy loss is evaluated.

**Figure 3:**
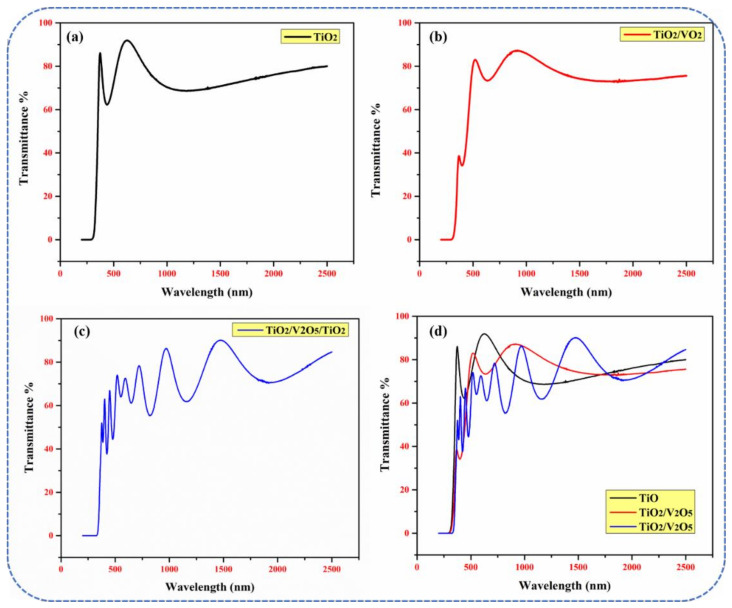
Transmittance spectra of (**a**) single-layer TiO_2_, (**b**) bi-layer TiO_2_/V_2_O_5_, and (**c**) multi-layer TiO_2_/V_2_O_5_/TiO_2_ thin films; (**d**) the combination of spectra for the mono-, bi-, and multi-layer thin films, respectively.

The optical properties (in particular, transmittance and the optical bandgap) of a material are crucial for determining its potential use as a solar water heater. The transmittance spectra of mono-, bi-, and tri-layers are shown in Figure 3 from 250 nm to 2500 nm. A transmittance of ~92%, ~82%, and ~78% was achieved in mono-, bi-, and tri-layers in the visible range, respectively, as shown in Figure 3a–d. The high transmittance for the mono-layer was due to its thinner overall film thickness compared to that of the bi- and tri-layers. After the deposition of the 50 nm-thick V_2_O_5_ layer (in the bi-layer), a substantial decrease of 10% was observed. This occurred because, as reported [37], the optical absorption edge of V_2_O_5_ thin films appears in the wavelength range from 400 nm to 500 nm (A_max_ = 390 nm; A_min_ = 330 nm). Thus, the absorption properties of V_2_O_5_ are relatively low at wavelengths > 500 nm. Moreover, the increased total thickness of the bi-layer (~200 nm) compared to that of the mono-layer (~150 nm) resulted in the decreased absorption of incident light. In the tri-layer, the decrease in the transmittance was two-fold due to: (i) an increase in the total thickness of the thin film (~600 nm) compared to the mono- and bi-layer thin films, and (ii) multiple incident photon reflections at the interface between the different TiO_2_/V_2_O_5_/TiO_2_/glass layers. In addition, a shift into the red region was present in the tri-layer due to the increased thickness of the thin film.

**Figure 4:**
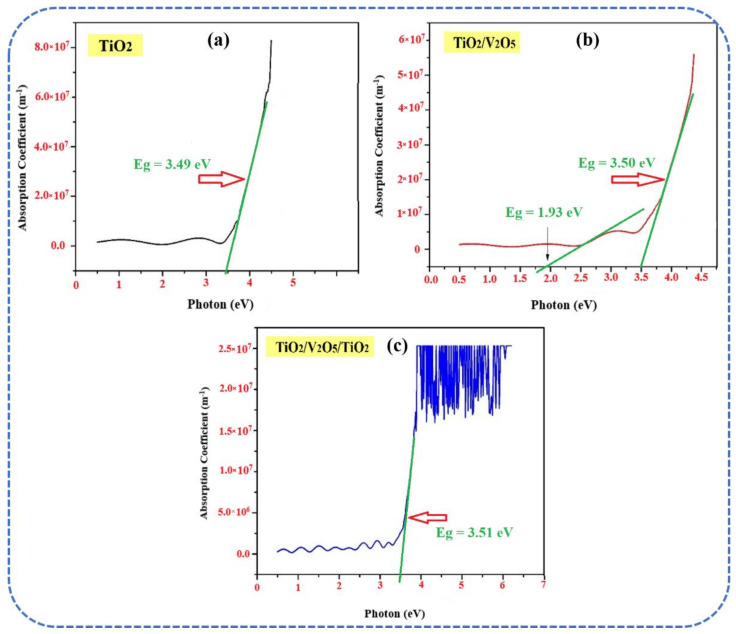
Plots of the optical absorption coefficient (α) vs. photon energy (hν) for (**a**) TiO_2_, (**b**) bi-layer TiO_2_/V_2_O_5_, and (**c**) multi-layer TiO_2_/V_2_O_5_/TiO_2_ thin films.

The optical absorption coefficient (α) was plotted as a function of photon energy (hν) and the spectra are shown in Figure 4a–c. Tauc’s plot was used to determine the optical bandgap (E_BG_) of the thin films. In the mono-layer, a typical E_BG_ ~ 3.49 eV for the anatase TiO_2_ (Figure 4a) was obtained [38]. Two E_BG_ values were observed in the bi-layer, which are attributable to the anatase TiO_2_ (E_BG_ ~ 3.50 eV) and α-V_2_O_5_ (E_BG_ ~ 1.93 eV), as shown in Figure 4b. The stoichiometry of V_2_O_5_ indicates that E_BG_ ~ 2.6 eV. Here, the decreased E_BG_ is due to the non-stoichiometry, oxide network, nanoporosity, and lower thickness of the V_2_O_5_ layer [39]. In the tri-layer, a single value of E_BG_ ~ 3.51 eV was observed in Figure 4c. The diffused E_BG_ is attributed to the inter-diffusion of the different layers during the deposition at relatively high substrate temperatures.

**Figure 5:**
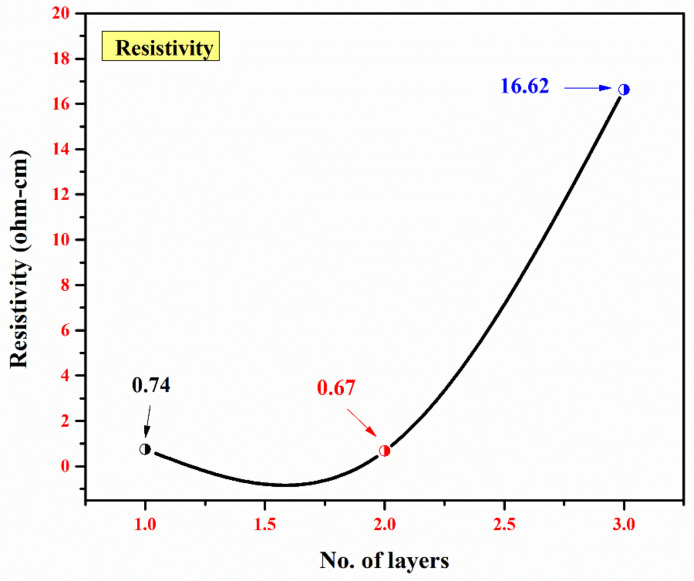
A plot of the no. of layers vs. resistivity (Ω-cm) for a single layer of TiO_2_, a bi-layer of TiO_2_/V_2_O_5_, and a multi-layer of TiO_2_/V_2_O_5_/TiO_2_.

Lastly, the resistivity of the mono-, bi-, and tri-layers is shown in Figure 5. First, the resistance was determined from the current (I) and potential (V). Subsequently, the resistivity was calculated by multiplying the resistance with the thickness (t) of the thin films. The conductivity was determined by taking the inverse of the resistivity, and the values are summarized in Table 2.

The resistivity of 0.74 Ω-cm was obtained in the mono-layer. A decrease in resistivity to 0.67 Ω-cm was observed in the bi-layer. Considering the optimal stoichiometry, the resistivity of a particular material depends on its morphological and structural parameters (e.g., thickness, phase composition, and dimensions). Bulk V_2_O_5_ has a resistivity of 2.1 × 10^7^ Ω-cm. As reported [40], a decrease in resistivity was observed for V_2_O_5_ thin films as their thicknesses were decreased, i.e., 8.7 × 10^6^ Ω-cm for 85 nm-thick films and 2.14 × 10^7^ Ω-cm for 112 nm-thick films. In the presented work, a substantial decrease in resistivity was observed and the reason for this is two-fold: (i) a relatively thin V_2_O_5_ layer (approx. 50 nm thick) and (ii) the TiO_2_/V_2_O_5_ interface. Moreover, the oxygen deficiency in the bi-layer resulted in an increase in the conductivity (1.48 Ω^−1^-cm^−1^ for the bi-layer compared to 1.35 Ω^−1^-cm^−1^ for the mono-layer). For the tri-layer, the conductivity decreased to 0.06 Ω^−1^-cm^−1^ due to the relatively thick top TiO_2_ layer (360 nm).

## 4. Conclusions

Smooth and uniform nanocomposite TiO_2_/V_2_O_5_/TiO_2_ multi-layer thin films were prepared as a solar water heater device using the electron beam evaporation technique. The total thickness of the SWHD thin film was approx. 600 nm on a 1 mm-thick transparent soda–lime glass substrate. The RBS and SEM analyses confirmed the successful formation of a nanocomposite multi-layered structure at the nanoscale level with optimal stoichiometry. The optical transmittance of the TiO_2_ mono-layer was ~92% in the visible region. In the TiO_2_/V_2_O_5_ bi-layer, the optical transmittance decreased to ~82% due to the increased total thickness (approx. 200 nm) compared to that of the mono-layer (150 nm) and due to the increased absorption of the incident light by V_2_O_5_. Nevertheless, the bi-layer possessed good transparency in the infrared region, contrary to the mono-layer. In the case of the TiO_2_/V_2_O_5_/TiO_2_ tri-layer, transmittances of ~78% in the visible and ~90% in the infrared regions were observed due to the increased total thickness of the thin film (approx. 600 nm) and multiple photon reflections between the constituent layers. An optical bandgap of approx. 3.5 eV was determined for the SWHD (tri-layer) with a high conductivity (resistivity of approx. 16.6 Ω-cm). 

## Figures and Tables

**Table 1:** Concentrations of mono-, bi-, and tri-layers as determined by RBS and EDS, and intended and determined thicknesses of the individual layers.

No. of Layers	Mono-Layer (TiO_2_)	Bi-Layer (TiO_2_/V_2_O_5_)	Tri-Layer (TiO_2_/V_2_O_5_/TiO_2_)
**Concentration determined by RBS**	Ti:30	Ti:18 V:8	Ti:35 V: 5
	O:70	O:74	O:60
**Intended thickness (nm)**	100	50	360
**Calculated thickness (nm)**	150	54	400
**Concentration determined by EDS (wt.%**)	Ti:18	Ti:20	Ti:42
	O:82	O:74	O:56
		V:6	V:2

**Table 2:** Resistance, resistivity, and conductivity of the mono-, bi-, and tri-layers.

Samples	Resistance (Ω)	Resistivity (Ω-cm)	Conductivity (Ω-cm)^−1^
mono-layer (TiO_2_)	504 × 10^6^	0.74	1.35
bi-layer (TiO_2_/V_2_O_5_)	336 × 10^6^	0.67	1.48
tri-layer (TiO_2_/V_2_O_5_/TiO_2_)	541 × 10^7^	16.62	0.06

## Data Availability

The data presented in this study are available on request from the corresponding author.
